# Primary Yolk Sac Tumor in the Cerebellar Hemisphere: A Case Report and Literature Review of the Rare Tumor

**DOI:** 10.3389/fonc.2021.739733

**Published:** 2021-11-05

**Authors:** Na Wu, Qiang Chen, Meng Chen, Jinbo Ning, Shulei Peng, Taotao Zhang, Wen Zhong, Song Duan, Chongjie Cheng, Yimin Xie

**Affiliations:** ^1^ Department of Pediatric Surgery/Pathology/Radiology/Sleep Medicine Center, Chongqing University Three Gorges Hospital, Chongqing, China; ^2^ Department of Neurosurgery, The First Affiliated Hospital of Chongqing Medical University, Chongqing, China

**Keywords:** yolk sac tumor, cerebellar hemisphere, endodermal sinus tumor, microsurgery, subtotal resection

## Abstract

Yolk sac tumor (YST) is one of rare malignant germ cell tumors (GCTs). Primary intracranial YST, also endodermal sinus tumor (EST), is a quite rare type of brain tumor. Here, we report a case of YST, review the relevant literature, and propose a treatment strategy for this rare tumor. A 6-year-old boy initially manifested symptoms of dizziness and vomiting. Computed tomography (CT) and magnetic resonance imaging (MRI) showed a large irregular oval tumor in the cerebellar hemisphere. We subtotally removed the tumor by microsurgery through the left suboccipital approach. Immunohistochemical staining showed that alpha fetoprotein (AFP) was positive and the Ki-67 proliferation index was high (60%), suggesting a germ cell tumor. After 3 months of follow-up, neither recurrence of tumor nor complications were found in the patient. The diagnosis of YST should be confirmed on the basis of clinical manifestations, neuroimaging and pathological findings. Gross total resection (GTR) is an ideal treatment for YST. However, due to the location of the tumor, GTR is usually difficult, and the rate of postoperative complications is high. This reported case shows that subtotal resection can be a good treatment strategy for YST.

## Background

Yolk sac tumor (YST), also known as endodermal sinus tumor (EST), is a rare central nervous system neoplasm because of the similarity of this tumor to the endodermal sinuses of the rat placenta described by Duval (1). Primary intracranial YSTs usually involve the pineal or suprasellar regions. Primary YST in the posterior fossa is extremely rare (2, 3). To our knowledge, only a few cases of primary YST were confined to the posterior fossa (2–14) (**Table 1**). Here, we report a case of YST in a patient who presented with dizziness and vomiting and review the diagnostic criteria and treatment strategies of this rare neural tumor.

**Table 1:** Brief review of yolk sac tumor cases of the posterior fossa in the literature.

Case No.	Authors & Year	Age	Sex	Tumor location	Treatment	Outcome
1	Arita et al. 1980 (4)	12	F	Pineal region	R	Death 13 months after diagnosis
2	Nakagawa et al. 1980 (8)	18	M	The fourth ventricle	S and R	Progress well in 3 months after diagnosis
3	Takeda et al. 1985 (12)	4	M	Left cerebellar hemisphere	S and R	Alive 19 months
4	Tajika et al. 1988 (11)	2.5	Unknown	Cerebellar hemisphere	Bs, S and C	Lack of information
5	Kirkove et al. 1991 (7)	14	M	Pineal region	S, C and R	Progress well in 24 months after the diagnosis
6	Tsukamoto et al. 1992 (13)	2	M	Cerebellar vermis	S, C and R	Death 30 months after diagnosis
7	Fujiwara et al. 1994 (6)	4	M	Right cerebellar hemisphere	S and C	Death 18 months after diagnosis
8	Nakase et al. 1994 (9)	5	M	Cerebellar vermis	S and C	Progress well, no recurrence, in 12 months after diagnosis.
9	Davidoff et al. 1996 (5)	16	M	Pineal region	S, C and R	Progress well, but died of radiation fibrosis 11 years later
10	Yang et al. 2003 (14)	2.5	M	Right cerebellar hemisphere	S, C and R	Alive 50 months
11	Cheon et al. 2006 (2)	3	M	Left cerebellar hemisphere	S and C	Progress well, no recurrence, in the 48 months after diagnosis.
12	Kuang et al. 2014 (3)	3	M	Left cerebellar hemisphere	S	Death 6 months after diagnosis
13	Shenoy et al. 2014 (10)	2	M	Cerebellar vermis	S	Lost to follow-up
14	Our case	6	M	Left cerebellar hemisphere	S and C	Progress well in 3 months after diagnosis→ ongoing follow-up

F, Female, M, Male, Bs, Biopsy, C, Chemotherapy, R, Radiotherapy, S, Surgery.

## Case Presentation

**Figure 1:**
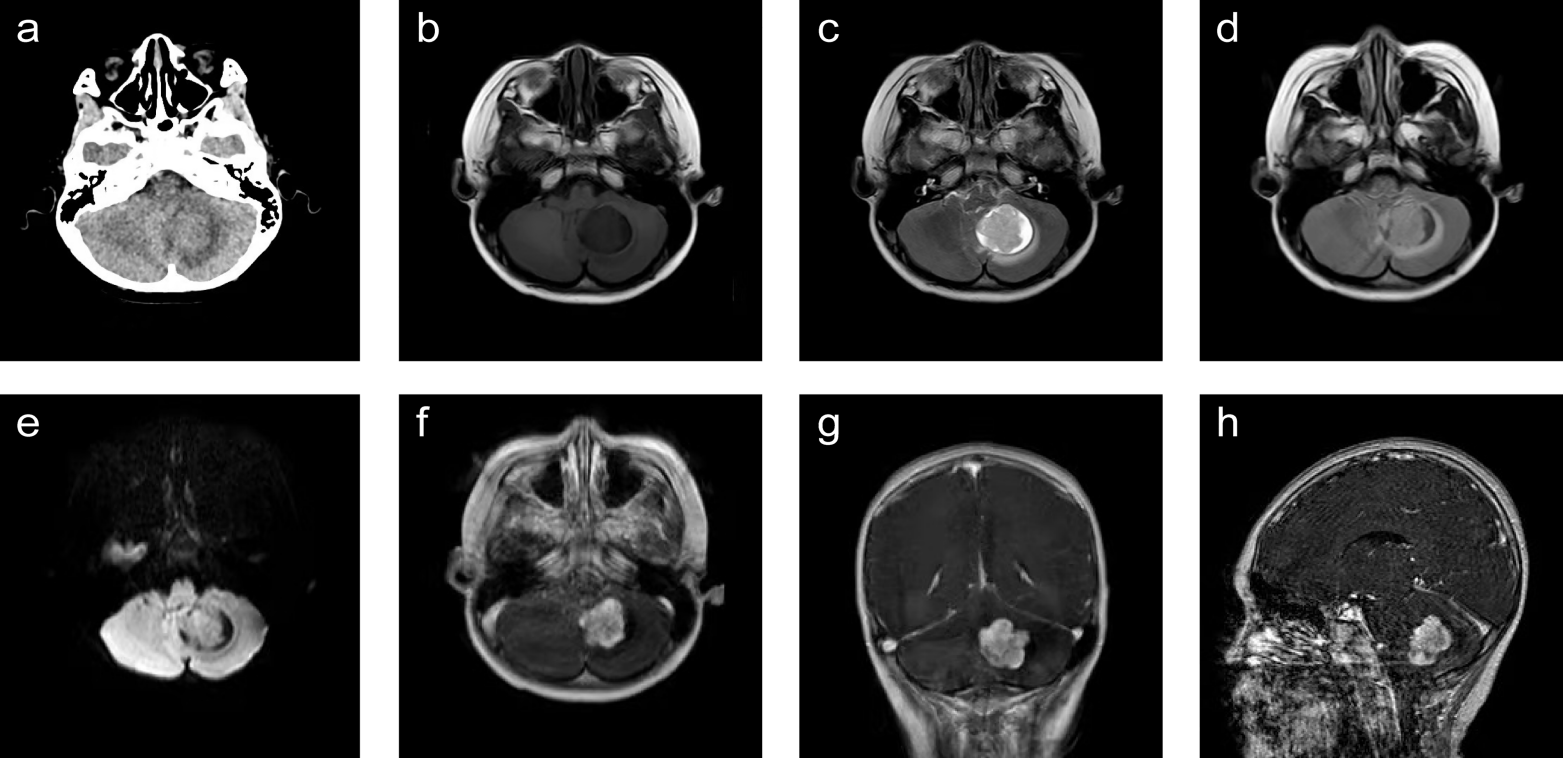
Radiological evaluation of yolk sac tumor before operation. A noncontrast CT scan showed a slightly hyperdense mass located in the left cerebellar hemisphere **(A)**. The tumor was slightly hypointense on axial T1-WI **(B)** and slightly hyperintense on axial T2-WI, FLAIR and DWI **(D, E)**. Postcontrast (gadolinium-enhanced) axial, coronal and sagittal MRI showed prominent homogeneous enhancement **(F‒H)**. The fourth ventricle was compressed and narrowed. Mild edema was noted around the lesion. **(B–H)**. WI, weighted imaging.

**Figure 2:**
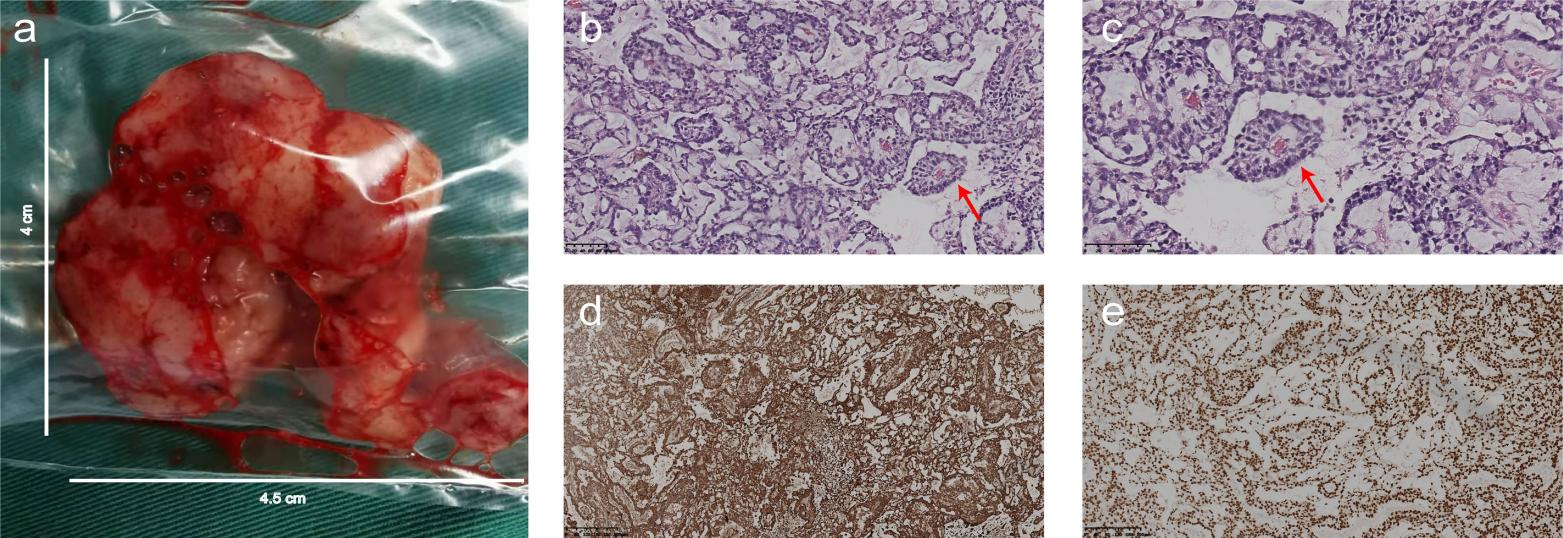
The operative findings and histopathology. **(A)** The tumor was located in the left cerebellar hemisphere and measured 4.5 cm× 4 cm. **(B, C)** Photomicrograph of hematoxylin and eosin staining showed that malignant tumor cells grew around the blood vessels or cavity, with typical Schiller-Duval bodies (red arrow). **(D)** Photomicrograph of immunohistochemical staining revealed that the majority of the tumor cells were strongly positive for alpha-fetoprotein (AFP) in the cytoplasm. **(E)** Photomicrograph of immunohistochemical staining suggested a positive reaction for Sal-like protein 4 (SALL-4) in the nucleus of the tumor cells. [Original magnifications: **(B, D, E)** 100×; **(C)** 200×].

**Figure 3:**
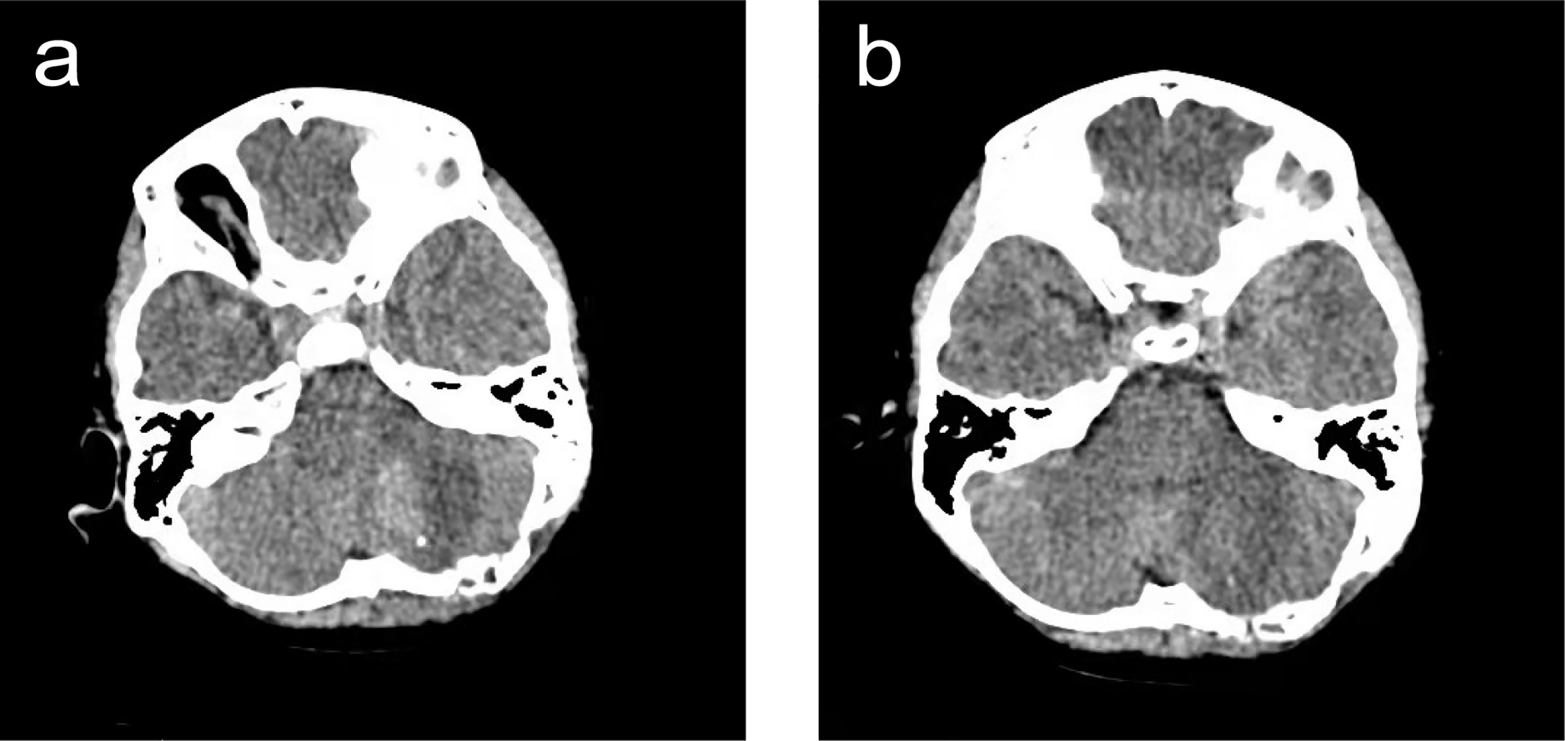
Radiological evaluation of YST post operation. CT scanning of the residual tumor on day 7 **(A)**. and two weeks after operation **(B)**. The size of the residual tumor significantly decreased. Patchy hyperdense shadow was seen in the left cerebellar hemisphere, surrounded by patchy low-density edema. No obvious abnormalities were found in the ventricle or cisterns. Displacement of middle structure was not found.

A 6-year-old boy was admitted to our hospital because of dizziness and vomiting for two weeks, without symptoms of convulsion and hemiplegia. The emotion and cognition of the patients were normal, and no obvious memory disorder was observed. Visual and aural problems were excluded. There was no abnormality in general physical examination or special neurological examination. Before operation, brain noncontrast computed tomography (CT) showed an irregular oval tumor of slight hyperdensity in the left cerebellar hemisphere (**Figure 1A**). Magnetic resonance imaging (MRI) showed an irregular ovoid mass with size of 3.6× 3.2 × 3.4 cm. The tumor showed slight hypointensity on axial T1-WI (**Figure 1B**) and slightly high signal intensity on axial T2-WI, FLAIR and DWI (**Figures 1C**–**E**). Postcontrast enhancement on axial, coronal and sagittal MRI indicated prominent homogeneous enhancement (**Figures 1F**–**H**). The fourth ventricle was compressed and narrowed. Mild edema was noted around the lesion. (**Figures 1B**–**H**). The surgery was performed *via* the left suboccipital approach on April 5, 2021. The intraoperative findings revealed a solid tumor in the left cerebellar hemisphere. The tumor was subtotally excised and measured to be 4.5 cm× 4.0 cm (**Figure 2A**). Histopathologic examination revealed that malignant tumor cells grew around the blood vessels or cavity, with typical Schiller-Duval bodies. Immunohistochemical staining was positive for alpha-fetoprotein (AFP), PCK, CK8, CD99 and Sal-like protein 4 (SALL-4) and negative for Vimentin, CK7, GFAP, S-100, CD34, FLI-1, CD99, CD30 and epithelial membrane antigen (EMA) (**Figures 2B**–**E**). The Ki-67 proliferation index was high (60%). Human chorionic gonadotropin-β and α-fetoprotein (AFP), serum tumor markers, were not measured, as a diagnosis of YST was not suspected at the preoperative stage. After operation, the AFP level in serum was 534.20 ng/ml, and human chorionic gonadotropin-β was negative. The patient recovered well after surgery. However, postoperative MRI was not performed because of poor cooperation. CT scanning of the residual tumor at 7 days and two weeks post operation showed that the tumor was significantly reduced (**Figure 3**). During 3 months of follow-up, the tumor did not recur, and the patient suffered no complications.

## Discussion

Yolk sac tumor (YST) is a rare malignant germ cell tumor (GCT) that usually originate from the gonad but is rarely observed extragonadally (15).

We retrieved studies published electronically between 01/01/1980 and 31/03/2021, including the articles published in English and Chinese journals and the English databases of PubMed, Google Scholar and WHO Site. For the first search, we used Chinese and English keywords of “yolk sac tumor or endodermal sinus tumor or YST or EST” and for the second retrieval, we used Chinese and English keywords of “posterior fossa or cerebellar hemisphere”. Reference articles of the published studies were also checked to retrieve more studies. One of the researchers evaluated the retrieval randomly to ensure no study had been excluded. Meanwhile, we corresponded with experts in this field to search for findings of unpublished studies, but failed.

Many studies have reported that YST occurs in the vagina, seminal vesicle, pancreas, omentum and stomach, as well as in the intracranial and sacrococcygeal regions (2, 3, 16–23). GCTs originating in the intracranial region almost always occur in the suprasellar regions or pineal gland, but tumors in the cerebellar hemisphere are extremely rare (2).

Histogenesis of extragonadal YST remains controversial. At present, there are two theories to explain the occurrence of primary extragonadal GCTs. One is the abnormal differentiation of somatic cells, the other is the malignant transformation of germ cell residues in the process of migration (16, 19). The latter may be more suitable to explain the YST location in our case.

Currently, no definite risk factors or syndromic neurological symptoms for YST are known, commonly presenting a variety of symptoms rather than a single manifestation are common. Our patient presented with dizziness and vomiting, which may have been attributed to the tendency of the tumor to distort the cerebellar hemisphere structure, causing intracranial hypertension.

It has been confirmed that the significant increase of AFP level in serum and cerebrospinal fluid is related to YST (3, 4). Initially, our case was not considered to be primary YST because it was consistent with medulloblastoma, given the age distribution and due to the rarity of YST in the cerebellar hemisphere. Therefore, the patient did not receive the preoperative test for serum AFP, but the final diagnosis was established by the pathology and the postoperative test for serum AFP (534.20 ng/ml).

Clinical diagnosis of YST requires imaging findings. Enhanced MRI is the best diagnostic imaging tool for evaluating brain tumors. YST is typically a well-defined round or oval tumor with isointense T1-weighted images and slightly hyperintense T2-weighted images (2, 3). After gadolinium injection, the tumor has strong homogenous enhancement (2, 3). The imaging findings of our patient were consistent with those in previous studies.

The typical morphological structure of YST is called the Schiller Duval body, which is composed of a monolayer of cubic or columnar neoplastic cells surrounding the capillaries, thin-walled blood sinus or small venous blood vessels. As a result, a vessel-centered and sleeve-shaped structure is formed, similar to a glomerulus-like structure (19, 24). We found a similar morphological structure in our case.

Studies have reported that AFP, Sal-like protein 4 and glypican-3 are sensitive diagnostic markers for YST (19, 24). The tumor in our case was positive for AFP and SALL-4. YST usually has high mitotic activity, with Ki-67 values typically near 60%. Our final diagnosis of YST was confirmed by microscopic pathology and immunohistochemical staining of the tumor. Furthermore, AFP is a useful marker not only for immunohistochemical staining and pathological diagnosis but also for evaluating treatment response and detecting recurrence (2, 3).

The prognosis of YST is usually poor because of its location in the deep brain structure, and gross total resection (GTR) is difficult without causing serious complications, while subtotal tumor resection (STR) is associated with high recurrence rates. The patients receiving GTR, compared with the patients receiving STR, had longer recurrence-free survival and better quality of life, but higher mortality and morbidity rates. We performed STR on the patient through the left suboccipital approach, which can adequately expose the tumor region with a less invasive parenchymal approach. The operation was successful, and the patient had no serious complications. Three months of follow-up showed no recurrence. Based on our experience of treating this case, we propose that STR could be an effective, safe and minimally invasive strategy for the treatment of YST patients.

YST is a subtype of germ cell tumor rarely located in the cerebellar hemisphere. The diagnostic criteria of YST include clinical manifestations, neuroimaging, and pathology. Surgical treatment is still critical for patients with YST; however, GTR may not be ideal when the tumor is adjacent to sensitive neurovascular structures. Therefore, treatment should be individualized. Our case report suggests that STR could be a good treatment strategy for YST.

In addition, previous studies reported surgical resection and adjuvant chemotherapy could improve life quality and prolong survival time in patients diagnosed with YST (1, 2, 4–9, 11, 13, 14, 25). During telephone follow-up, we learned that the patient had undergone chemotherapy at another hospital after discharge from our hospital, which may also contribute to the recovery of our patient. Because of the rarity of cerebellar YST, the surgical approach and treatment strategy were chosen based on previous studies. Given the short follow-up period, we cannot determine whether the patient was completely cured. The case will be continuously followed up, and information on disease status and survival will be collected. According to the previous literature and our case, we believe that surgical resection and adjuvant chemotherapy may result in prolonged survival.

## Author Contributions

NW and QC designed the case report. YX, MC, JN, and TZ all participated in the operation and management of the patient. NW, CC, and YX reviewed the literature and drafted the article. WZ, SP, and SD prepared radiological and histology figures and provided immunohistochemical analysis. All authors contributed to the article and approved the submitted version.

## Conflict of Interest

The authors declare that the research was conducted in the absence of any commercial or financial relationships that could be construed as a potential conflict of interest.

## Publisher’s Note

All claims expressed in this article are solely those of the authors and do not necessarily represent those of their affiliated organizations, or those of the publisher, the editors and the reviewers. Any product that may be evaluated in this article, or claim that may be made by its manufacturer, is not guaranteed or endorsed by the publisher.
